# Mining RNA-Seq Data to Depict How *Penicillium digitatum* Shapes Its Transcriptome in Response to Nanoemulsion

**DOI:** 10.3389/fnut.2021.724419

**Published:** 2021-09-14

**Authors:** Ruopeng Yang, Xiu Chen, Qiang Huang, Chuying Chen, Kannan R. R. Rengasamy, Jinyin Chen, Chunpeng (Craig) Wan

**Affiliations:** ^1^Jiangxi Key Laboratory for Postharvest Technology and Nondestructive Testing of Fruits & Vegetables, College of Agronomy, Jiangxi Agricultural University, Nanchang, China; ^2^College of Life Science and Technology, Honghe University, Mengzi, China; ^3^Green Biotechnologies Research Centre of Excellence, University of Limpopo, Mankweng, South Africa; ^4^College of Materials and Chemical Engineering, Pingxiang University, Pingxiang, China

**Keywords:** citrus, RNA-seq, *Penicillium digitatum*, nanoemulaion, antifungal

## Abstract

*Penicillium digitatum* is the most severe pathogen that infects citrus fruits during storage. It can cause fruit rot and bring significant economic losses. The continuous use of fungicides has resulted in the emergence of drug-resistant strains. Consequently, there is a need to develop naturally and efficiently antifungal fungicides. Natural antimicrobial agents such as clove oil, cinnamon oil, and thyme oil can be extracted from different plant parts. They exhibited broad-spectrum antimicrobial properties and have great potential in the food industry. Here, we exploit a novel cinnamaldehyde (CA), eugenol (EUG), or carvacrol (CAR) combination antifungal therapy and formulate it into nanoemulsion form to overcome lower solubility and instability of essential oil. In this study, the antifungal activity evaluation and transcriptional profile of *Penicillium digitatum* exposed to compound nanoemulsion were evaluated. Results showed that compound nanoemulsion had a striking inhibitory effect on *P. digitatum* in a dose-dependent manner. According to RNA-seq analysis, there were 2,169 differentially expressed genes (DEGs) between control and nanoemulsion-treated samples, including 1,028 downregulated and 1,141 upregulated genes. Gene Ontology (GO) analysis indicated that the DEGs were mainly involved in intracellular organelle parts of cell component: cellular respiration, proton transmembrane transport of biological process, and guanyl nucleotide-binding molecular function. KEGG analysis revealed that metabolic pathway, biosynthesis of secondary metabolites, and glyoxylate and dicarboxylate metabolism were the most highly enriched pathways for these DEGs. Taken together, we can conclude the promising antifungal activity of nanoemulsion with multiple action sites against *P. digitatum*. These outcomes would deepen our knowledge of the inhibitory mechanism from molecular aspects and exploit naturally, efficiently, and harmlessly antifungal agents in the citrus postharvest industry.

## Introduction

During the postharvest, handling, and transportation process, citrus usually suffers significant losses. Related studies show that citrus postharvest rot is mainly green mold caused by *P. digitatum*, accounting for more than 80% of the total crop losses (1). Currently, chemical agent application is the chief approach to combat this pathogen; notwithstanding it has a specific effect of prolonging the storage period of citrus, chemical agents have aroused concern about resistant strains, human health, and environmental pollution (2). Searching for exploitation of natural antifungal substances from plants as chemical compound alternatives has attracted more attention of researchers (3–7). Essential oil, a volatile aromatic compound, can be extracted from different parts of the plant (8). Essential oils have been recognized as GRAS (generally recognized as safe) by the US food and drug administration. Cinnamaldehyde and eugenol are the main components of cinnamon essential oil and clove oil, respectively. Carvacrol was found in the thyme oil or oregano oil and has been reported to possess antimicrobial activities (9–11). Cinnamaldehyde could effectively inhibit *Aspergillus flavus* through plasma membrane damage (5). Carvacrol has been demonstrated to have an antibacterial efficiency against *Listeria monocytogenes* and *Escherichia coli* (12); eugenol has an antifungal activity against *Cryptococcus gattii* and *C. neoformans* (13).

Antifungal drug combination against fungal growth is a reasonable strategy. Compared with individual fungicides, compound fungicides can reduce the effective dose. When two or more drugs act synergistically, it can reduce the cost and potential toxicity (14, 15). The combination can delay the evolution of fungal resistance. More importantly, they can make existing and approved drugs repurposed, bypassing the expensive and lengthy development of new antifungal agents (16, 17). Essential oils encapsulated in nanoform are a promising alternative for antimicrobial strategy (18, 19). Nanoemulsion can enhance an antibacterial or antifungal activity of essential oil against microorganisms and overcome the drawbacks of essential oils, such as low solubility, instable under light or oxygen (20, 21).

Transcriptomics is used to illuminate the transcriptional level of genes under different stress to elucidate its regulatory mechanism. In particular, a new high-throughput sequencing method (RNA-seq) has been widely used to study eukaryotic transcriptomes (22–24). RNA-seq technology has the advantages such as high-throughput, high-sensitivity, digital signal, accurate results, good reproducibility, low cost, and no species limitation. It can accurately explain the pathogenic mechanism of fungi and the inhibition mechanism of antifungal substances on microorganisms at the molecular level (25, 26).

In this study, the antifungal mechanism of a combination of eugenol, cinnamaldehyde, and carvacrol nanoemulsion on *P. digitatum* was determined with an RNA-Seq approach. *P. digitatum* transcriptome and the DEGs between nanoemulsion-treated and untreated samples were obtained, which provide a theoretical reference for improving the prevention and control effect of citrus postharvest diseases.

## Materials and Methods

### Reagent and Fungal Cultivation

Cinnamaldehyde, citral, and eugenol were purchased from Aladdin Co., Ltd. (Shanghai, China). First, a combination of cinnamaldehyde, citral, and eugenol in a ratio of 1:1:1 was dissolved in 2% Tween-80, then blended with a high-shear mixer (ULTRA TURRAX^®^ T18 digital, IKA, Staufen, Germany), and finally passed through a microfluidizer (M-110P, Microfluidics Corporation, United States) to obtain a stock solution of 100 mg/ml. *P. digitatum* was isolated from green mold-infected citrus in our laboratory and maintained on PDA medium at 4°C. Fungal conidia from a 7-day-old culture were washed with sterilized water, filtered through four layers of gauze, and finally adjusted to a suspension of 1 × 10^7^ conidia/mL by hemocytometer.

### Antifungal Activity of Compound Nanoemulsion

The antifungal activity of compound nanoemulsion against *P. digitatum* was evaluated by the mycelial growth inhibition method (27) with some modifications. The stock nanoemulsions were added to the non-coagulated PDA to obtain the final concentration range (0.25, 0.125, 0.0625, 0.0313, 0.0156, and 0.0078 mg/mL). Two percent Tween-80 was added to PDA and taken as control. The plugs of mycelia (6 mm diameter) from the activated *P. digitatum* were transformed to the center of PDA plates and incubated at 28 ± 2°C for 7 days in the dark. The following formula calculated the growth inhibition rates of samples: mycelia growth inhibition rate = (control colony diameter-treated colony diameter) / (control colony diameter) × 100%. There are three replicates of each concentration, and the experiment was conducted twice.

### Preparation of Nanoemulsion Treatment

About 1.5 ml spore suspension (1 × 10^7^ conidia/ml) was added to 150 ml liquid culture medium (potato 200 g, glucose 20 g, 1 L distilled water) and cultured in an incubation shaker at 140 rpm for 48 h. Then, the mycelia were centrifuged at 3,000 × *g* for 20 min, followed by washing with phosphate buffer (pH 7.0) three times and resuspended in 100 ml PBS (pH 7.0). Afterward, the stock nanoemulsion was added to the flask (D-T) to the final concentration of 0.125 mg/mL, which is the minimum inhibitory concentration on *P. digitatum*, and then kept in an incubation shaker for 12 h; there was not nanoemulsion added, which was taken as the control (D-C). Finally, the mycelia, which removed phosphate buffer, were rapidly frozen in liquid nitrogen and kept in a −80°C refrigerator. Each treatment was performed three times.

### Extraction, Quantification, and Qualification of RNA

Total RNA preparation, quality control, cDNA libraries construction, and RNA-seq were conducted by Shanghai Applied Protein Technology (APT) Co., Ltd. The TRIzol reagent was used (Invitrogen, United States) to isolate total RNA according to the instruction. One percent agarose gel electrophoresis was used to evaluate RNA degradation and contamination. RNA purity, concentration, and integrity were checked using the NanoPhotometer^®^ spectrophotometer (IMPLEN, CA, United States), Qubit^®^ RNA Assay Kit in Qubit^®^ 2.0 Fluorometer (Life Technologies, CA, United States), RNA Nano 6000 Assay Kit of the Bioanalyzer 2100 system (Agilent Technologies, CA, United States) respectively. Each group were conducted for triple biological replicates.

### Library Preparation for Transcriptome Sequencing

The input material is 3 μg RNA of each sample for the RNA preparations. Briefly, mRNA was purified from total RNA using poly-T oligo-attached magnetic beads by using NEBNext^®^ UltraTM RNA Library Prep Kit for Illumina^®^ (NEB, United States) following the manufacturer's recommendations, and each sample was to attribute sequences by adding index codes. The divalent cations under elevated temperature in NEBNext First Strand Synthesis Reaction Buffer (5X) were used to fragment. The fragments were used to synthesize the first-strand cDNA with random hexamer primer and M-MuLV Reverse Transcriptase (RNase H). The second-strand cDNA was transformed from the first-strand cDNA using RNase H and DNA polymerase I. Fragments of preferential lengths of about 250~300 bp were purified using the AMPure XP system (Beckman Coulter, Beverly, United States), end-repaired, and adapter-ligated. Then, 3 μl of USER Enzyme (NEB, United States) was used with size-selected, adaptor-ligated cDNA at 37°C for 15 min followed by 5 min at 95°C before PCR. Then, PCR was performed with Phusion High-Fidelity DNA polymerase, universal PCR primers, and index (X) primer. Finally, PCR products were purified (AMPure XP system), and library quality was assessed on the Agilent Bioanalyzer 2100 system.

### *De novo* Transcriptome Assembly

After filtration of the lower-quality reads, obtained clean reads were used for the following analysis. The clean reads were mapped to the reference genome and assembled according to the previous method (Ouyang et al., 2016). The Trinity program was used to *de novo* assemble processed reads (28). The sequencing reads were used to construct a k-mer graph (*k* = 25). The seed k-mers were extended to both ends to form a contig. The overlapped contigs are clustered to form components, and each component becomes a set of possible representations of variable splicing isoform or homologous genes. Each component has a corresponding de Bruijn graph. The de Bruijn graph is simplified, and the path with continuous nodes is combined to form a more extended sequence, and the best path is found to obtain the transcriptional sequence.

### Annotations of Unigenes

First, we were blasting the optimal transcript against the NCBI non-redundant protein sequences database (NR) and Swiss-Prot to obtain unigenes. To further annotate the unigenes, the Blast2GO program with Blast2GO default parameters was used to obtain Gene Ontology (GO) annotations. GO enrichment analyses of DEGs were implemented by the cluster profile package (version 3.4.4), in which gene length bias was corrected. GO terms with corrected *P*-value < 0.05 were considered significantly enriched by DEGs. We used the clusterProfiler package (version 3.4.4) to annotate the pathways of DEGs in KEGG pathways.

### Quantification of Gene Expression Level

The FeatureCounts software (version 1.5.0-p3) was used to count the reads numbers mapped to each gene. Expression levels of unigenes were normalized and calculated as the values of fragments per kilobase of transcripts per million mapped fragments (FPKM). To select DEGs by DESeq2 package (version 1.16.1), DESeq2 provides statistical routines for determining differential expression in digital gene expression data using a model based on the negative binomial distribution. The resulting *P*-values, which are based on the negative binomial distribution model, false discovery rate was calculated by Benjamini and Hochberg's approach. Genes with an adjusted *P*-value < 0.05 were assigned as differentially expressed.

### Validation of RNA-Seq by qRT-PCR

Eight DEGs were selected randomly to confirm the RNA-Seq data by qRT-PCR. The total RNA was reverse-transcribed with a Hifair^®^ III 1st Strand cDNA Synthesis SuperMix for qPCR (gDNA digester plus) (YEASEN Biotech Co, Ltd. Shanghai, China), according to the manufacturer's instructions. The qRT-PCR assay was performed on CFX Real-Time PCR Detection Systems (CFX96, BIO-RAD, United States). The actin gene was obtained as an internal reference (29), and other target gene primer pairs were designed by Primer-Blast of NCBI (National Centre of Biotechnology Information, Bethesda, MD, United States). The amplification program was as follows: one cycle at 95°C for 3 min, and 39 cycles at 95°C for 5 s, 60°C for 3 min. The relative gene expression was analyzed according to the method described in the study by Livak and Schmittgen (30). The primers for the qRT-PCR were synthesized by Tsingke Biotech (Beijing, China) and are presented in Table 1. Each reaction was conducted three times.

**Table 1:** Primers of eight DEGs used for gene expression analysis by RT-qPCR.

**Gene ID**	**Primer**	**Sequence (5'-3')**
TR1589_c0_g1	Forward Reverse	5'-TCAACTTCAGGCTCGACACC-3' 5'-GTCTCGCACACGGGATACAA-3'
TR4477_c0_g1	Forward Reverse	5'-ACGGCAGAAGGGCTAAGTTC-3' 5'-AAGGCTACAATGCGAGGTCC-3'
TR971_c0_g1	Forward Reverse	5'-TAGCATGACGCTGACACGTT-3' 5'-AGGAATCACAAGGCGTGGAG-3'
TR1461_c0_g1	Forward Reverse	5'-CTCACGCGATGGCTACAGAT-3' 5'-TCATCGCCTGAACCACTTCC-3'
TR124_c0_g2	Forward Reverse	5'-ATAGCCATCTGTGCGGTAGC-3' 5'-CACCCTCTGACCTACTCCGA-3'
TR2819_c0_g1	Forward Reverse	5'-CCAGCACCAACAGCCATCTA-3' 5'-GTTCGGTGGGGAATGGGAAT-3'
TR283_c0_g1	Forward Reverse	5'-CCAGACGAGGGACTTGATACC-3' 5'-CGGTCTGCCTGCTGAGATTG-3'
TR38_c0_g2	Forward Reverse	5'-TGTTCTCTCGTTCCGCCAAA-3' 5'-GAGAGGATATGGGTGGTGCG-3'
Actin	Forward Reverse	5'-TGCGCTGAACCGAACTGCCG-3' 5'-TCGGGAGCCTCGAAGCGCTC-3'

### Determination of Oxidative Parameters

After the treatment with nanoemulsion, the mycelia were collected and used to determine the membrane lipid peroxidation-related parameters. The malondialdehyde (MDA) content was determined by following the previous method with minor modifications (31). According to the manufacturer's instructions, the H_2_O_2_ content was determined according to the method described in the study by Song et al. (32), using an H_2_O_2_ detection kit (Nanjing Jiancheng, Nanjing, China).

### Determination of Soluble Protein Contents

Coomassie Bright Blue method was used to determine the change in soluble protein content in the mycelium of *P. digitatum*, and bovine serum protein was used as the standard curve. *P. digitatum* was incubated in a shaker at 27°C, 180 rpm for 48 h. The exact amounts of mycelia were suspended in 10 mL phosphoric acid buffer (pH 7.0), and nanoemulsion was added to make the final concentration of 0.125 mg/mL. The culture was kept in a shaker at 27°C, 180 rpm, for 0, 2, 4, 6, and 12 h. The mycelium was frozen by liquid nitrogen, and then the mycelia were grounded into a paste with 5 mL distilled water and centrifuged at 4°C, 12,000 rpm for 15 min. About 1.0 mL of supernatant was taken, and 5 mL of Coomassie Bright Blue solution was added. The solution was shaken thoroughly, then let stand for 5 min. The OD value was measured at 595 nm with distilled water as blank control. According to the standard curve, the soluble protein content of mycelia was calculated, and the result was expressed as the mass of soluble protein per gram of mycelia (mg/g). Each treatment has three biological replicates, and the experiment was conducted twice.

### Statistical Analysis

Data were expressed as the mean ± SD, which was conducted triplicate, and were performed using SPSS 22.0 (SPSS Inc., United States). One-way ANOVA and Duncan's multiple range test were used to evaluate the significance (*p* < 0.05).

## Results

### Antifungal Activity of Nanoemulsion

**Figure 1:**
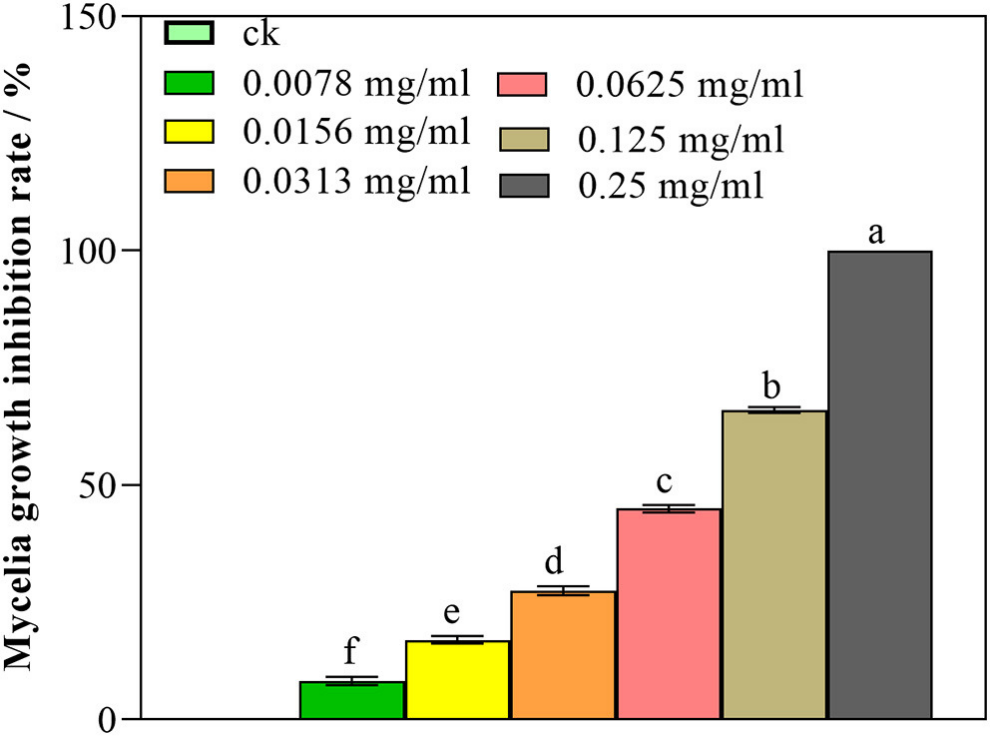
Antifungal activity of nanoemulsions against *P. digitatum*. Values are the mean ± standard deviation (SD) of three replicates. Different letters above the bars on columns indicate a significant difference (*P* < 0.05).

As illustrated in Figure 1, nanoemulsion showed an increased inhibitory activity against *P. digitatum* with increasing concentration. At the concentration of 0.125 mg/mL, the mycelia germination inhibition rate was 66.05%; furthermore, while 0.25 mg/mL concentration significantly inhibited the mycelia growth, and the inhibition rate was 100.00% (*p* < 0.05) on the 7th day, the control did not show an inhibitory activity. These results indicated that nanoemulsion could inhibit the mycelia growth of *P. digitatum* in a dose-dependent manner.

### Transcriptome Sequencing Quality

The quality inspection was conducted on all samples to meet the requirements of sequencing database construction. From the sequencing data shown in Table 2, RNA sequencing of nanoemulsion-treated and untreated *P. digitatum* generated 46.9 and 42.7 million raw reads, respectively. After filtering the adaptor sequences, 46.7 and 42.5 million clean reads were obtained. The GC content of each sample is above 50%, indicating that the base content is stable and there was no AT or GC separation; the Q30 base percentage of all samples is higher than 95%, which means obtained clean reads were accurate and can be used for subsequent analysis.

**Table 2:** RNA-seq data in control (D-C) and treatment (D-T) of *P. digitatum*.

**Parameter**	**D-C**	**D-T**	**D-C and D-T**
Raw reads	46,993,647	42,705,338	
Clean reads	46,761,640	42,470,344	
Total mapped	86.38%	88.75%	
Clean bases	6.48 G	5.89 G	
Q20 (%)	98.38	98.45	
Q30 (%)	95.2	95.1	
GC content (%)	52.06	51.31	
The number of all genes			18,113
Genes annotation against GO			1,627
DEGs annotation against GO			1,020
Genes annotation against KEGG			5,787
DEGs annotation against KEGG			249
Up-regulated genes			1,141
Down-regulated genes			1,028

### Transcriptional Stress Response of *P. digitatum* to Nanoemulsion

**Figure 2:**
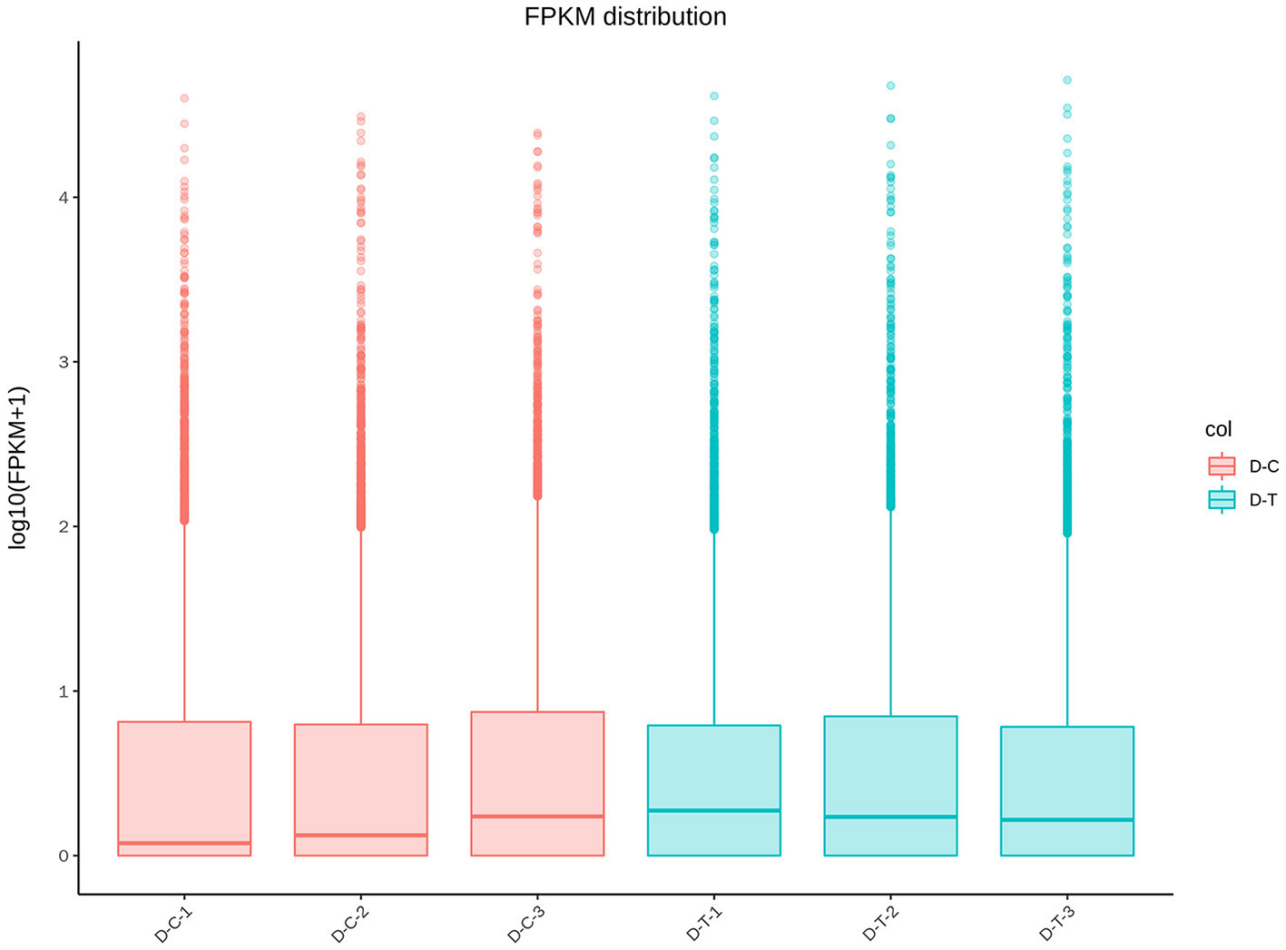
Gene expression-level comparison between control and nanoemulsion treatment: **(A)** FPKM distribution (Fragments Per Kilobase of transcript sequence per Millions of base pairs sequenced) and **(B)** FPKM density distribution.

**Figure 3:**
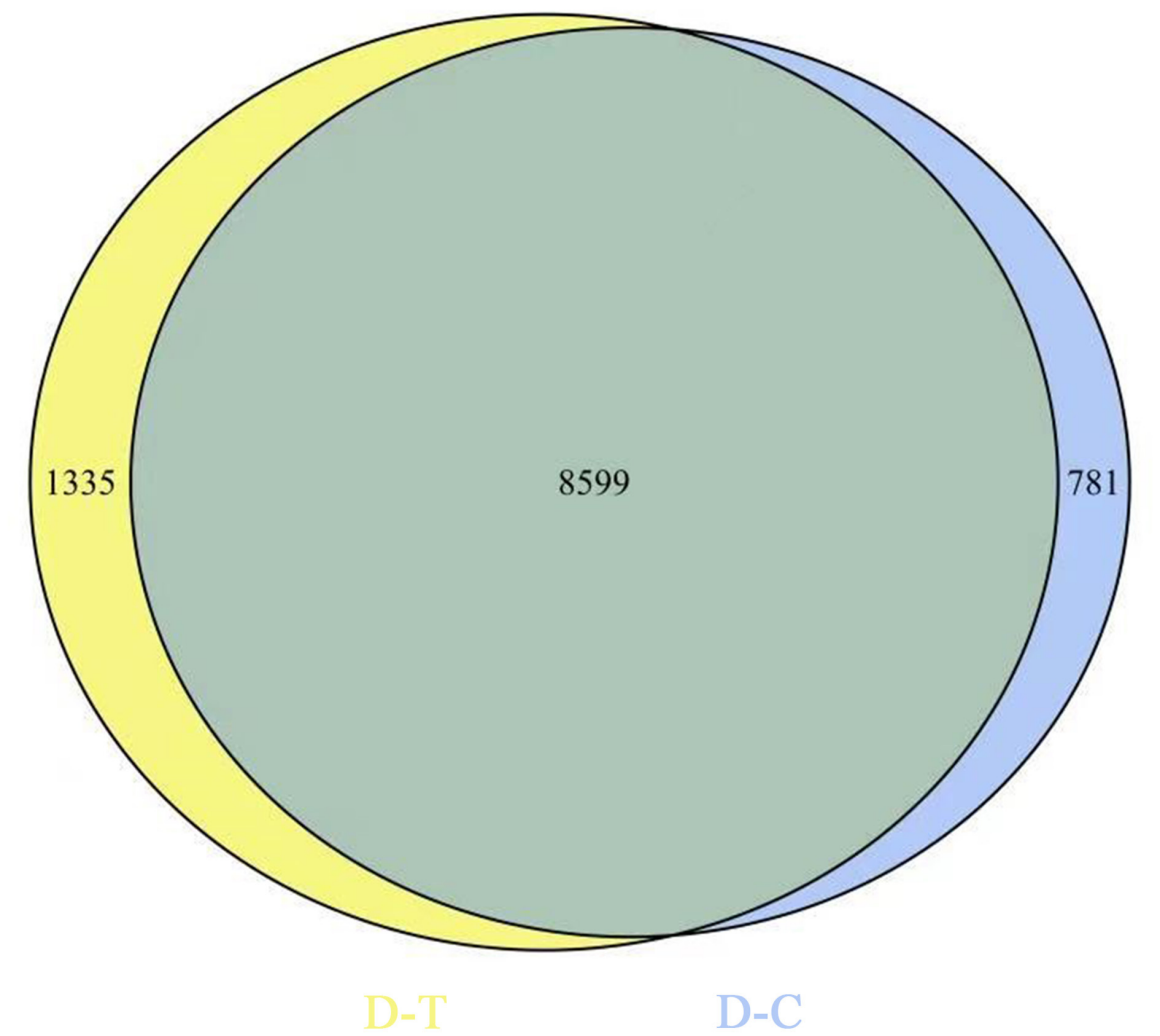
Venn diagram of gene expression. The number in the blue circle and yellow circle indicates the total number of genes expressed in the control and nanoemulsion treatment samples, respectively, and the green part of circles indicates that the gene is co-expressed in both samples.

**Figure 4:**
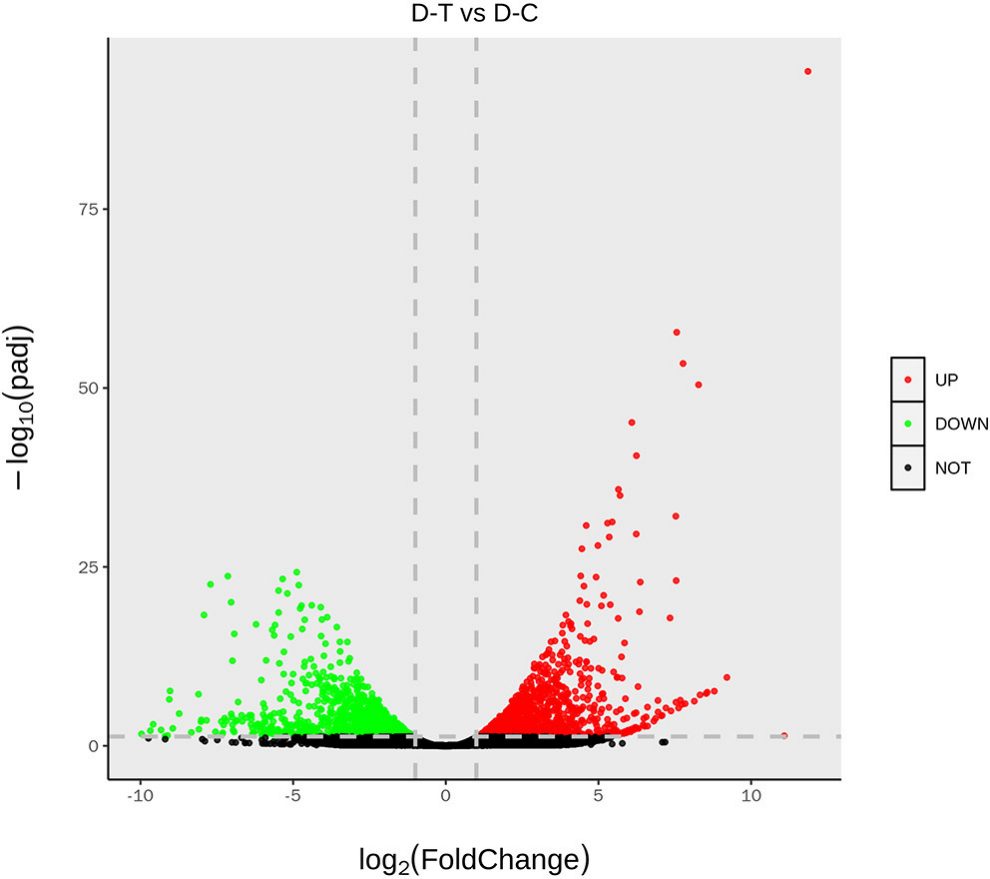
The volcano of differentially expressed genes. The abscissa indicates gene expression in different treatments; the ordinate indicates the statistical significance of gene expression. The scatter in the graph represents each gene, the black dot represents the gene with no significant difference, the red dot represents the upregulated gene with a significant difference, and the green dot represents the downregulated gene with a significant difference.

Figure 2 represents gene expression distribution differences and density distribution in control and nanoemulsion treatment samples. The gene expression distribution is different in three biological replicates from the same treatment, which is revealed in Figure 2A. Figure 2B indicates that most gene expression is in the lower level, while a few genes expression is in the higher level. Figure 3 shows 9,380 and 9,934 genes expressed in control and nanoemulsion treatment, of which 8,599 genes were co-expressed. The volcano plots revealed the difference in gene expression level and statistically significant difference in the DEGs. Two thousand one hundred and sixty-nine genes were differentially expressed in *P. digitatum* after nanoemulsion treatment: 1,141 genes showed upregulation tendency, whereas 1,028 genes were downregulated (Figure 4).

### Enrichment Analysis of GO and KEGG Pathways

**Figure 5:**
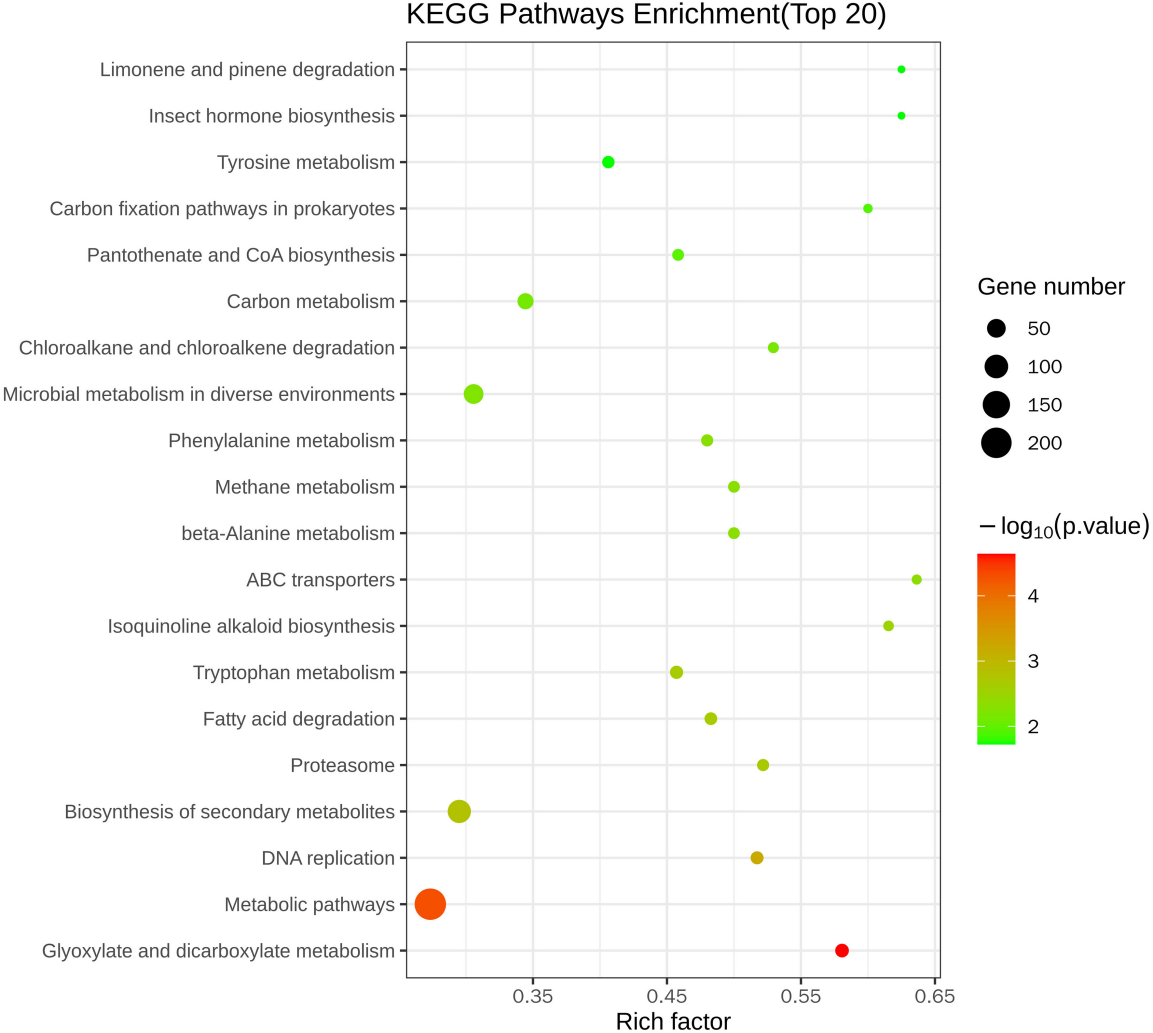
Top 20 KEGG enrichment pathways of differentially expressed genes.

**Figure 6:**
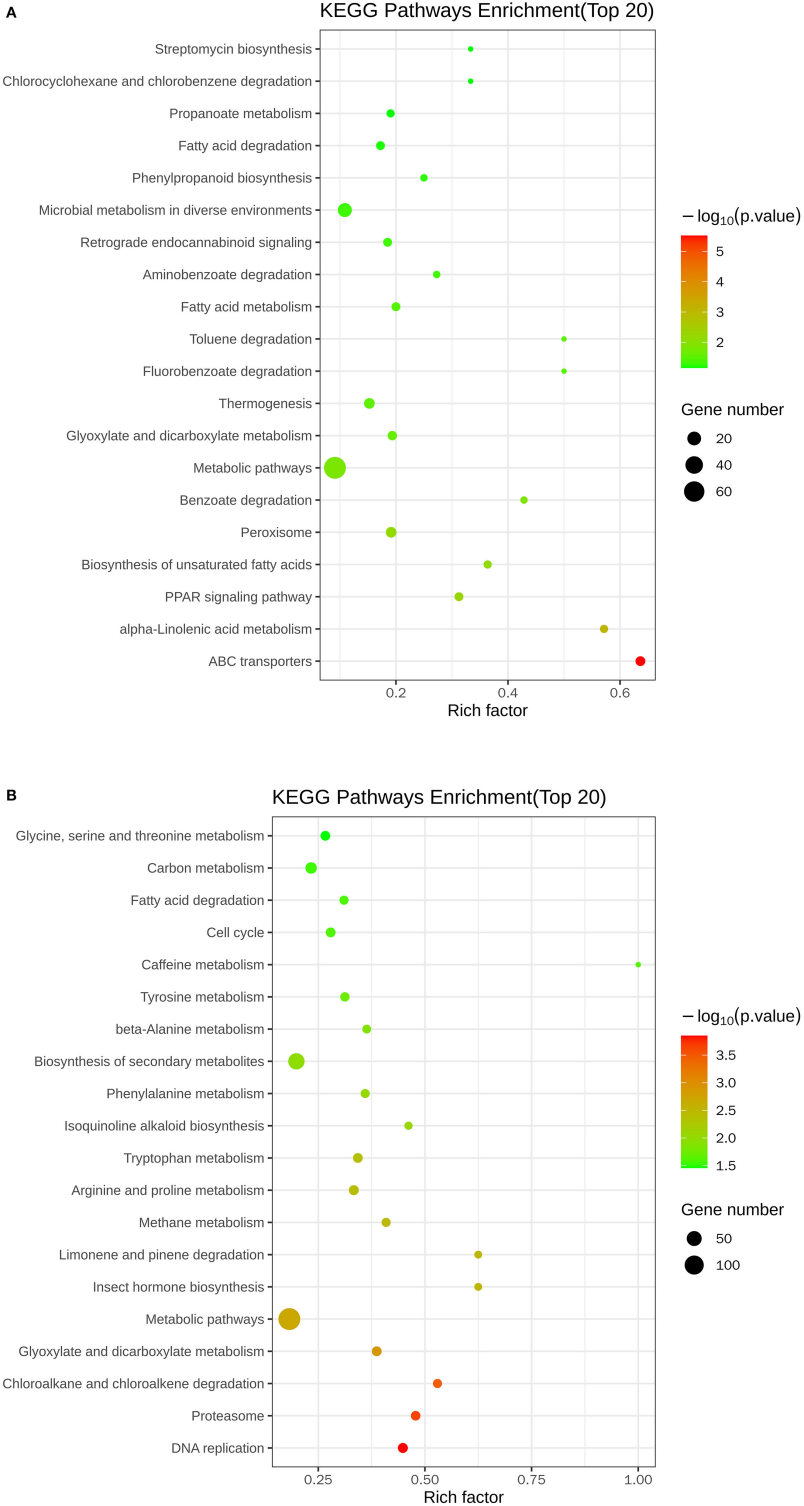
Scatter plots of the top 20 KEGG enrichment of upregulated **(A)** and downregulated **(B)** DEGs between control and nanoemulsion treatment.

Figure 5 and Table 3 illustrate the most enriched biochemical pathways mapped by DEGs revealed by KEGG pathway analysis (*P*-value < 0.05). Therein, the significantly abundant DEGs (216) were enriched in the metabolic pathway (ko01100), 95 DEGs were enriched in the biosynthesis of secondary metabolites (ko01110), 18 DEGs were enriched in glyoxylate and dicarboxylate metabolism (ko00630), and 15 and 14 DEGs were enriched in DNA replication (ko03030) and fatty acid degradation (ko00071), respectively. Figures 6A,B shows the top 20 upregulated and downregulated DEGs in KEGG enrichment (*P*-value < 0.05). The results indicated that the downregulated pathways involved mainly belonged to the DNA replication, proteasome, chloroalkane, and chloroalkene degradation. The upregulated pathways involved are ABC transporters, alpha-linolenic acid metabolism, metabolic pathways, and biosynthesis of unsaturated fatty acids.

**Table 3:** Mostly enriched KEGG pathway of DEGs in *P. digitatum*.

**Pathway**	**Input**	**Background**	**Pathway**
	**number**	**number**	**ID**
Glyoxylate and dicarboxylate metabolism	18	31	ko00630
Metabolic pathways	216	790	ko01100
DNA replication	15	29	ko03030
Biosynthesis of secondary metabolites	95	322	ko01110
Fatty acid degradation	14	29	ko00071
Proteasome	12	23	ko03050
Tryptophan metabolism	16	35	ko00380
Isoquinoline alkaloid biosynthesis	8	13	ko00950
ABC transporters	7	11	ko02010
beta-Alanine metabolism	11	22	ko00410
Methane metabolism	11	22	ko00680
Phenylalanine metabolism	12	25	ko00360
Microbial metabolism in diverse environments	59	193	ko01120
Chloroalkane and chloroalkene degradation	9	17	ko00625
Carbon metabolism	31	90	ko01200
Pantothenate and CoA biosynthesis	11	24	ko00770
Carbon fixation pathways in prokaryotes	6	10	ko00720
Insect hormone biosynthesis	5	8	ko00981
Limonene and pinene degradation	5	8	ko00903
Tyrosine metabolism	13	32	ko00350
Lysine degradation	11	26	ko00310
Aminobenzoate degradation	6	11	ko00627
Cyanoamino acid metabolism	8	18	ko00460
Naphthalene degradation	7	15	ko00626
Nitrogen metabolism	7	15	ko00910
Linoleic acid metabolism	3	4	ko00591
Arginine and proline metabolism	14	39	ko00330

**Figure 7:**
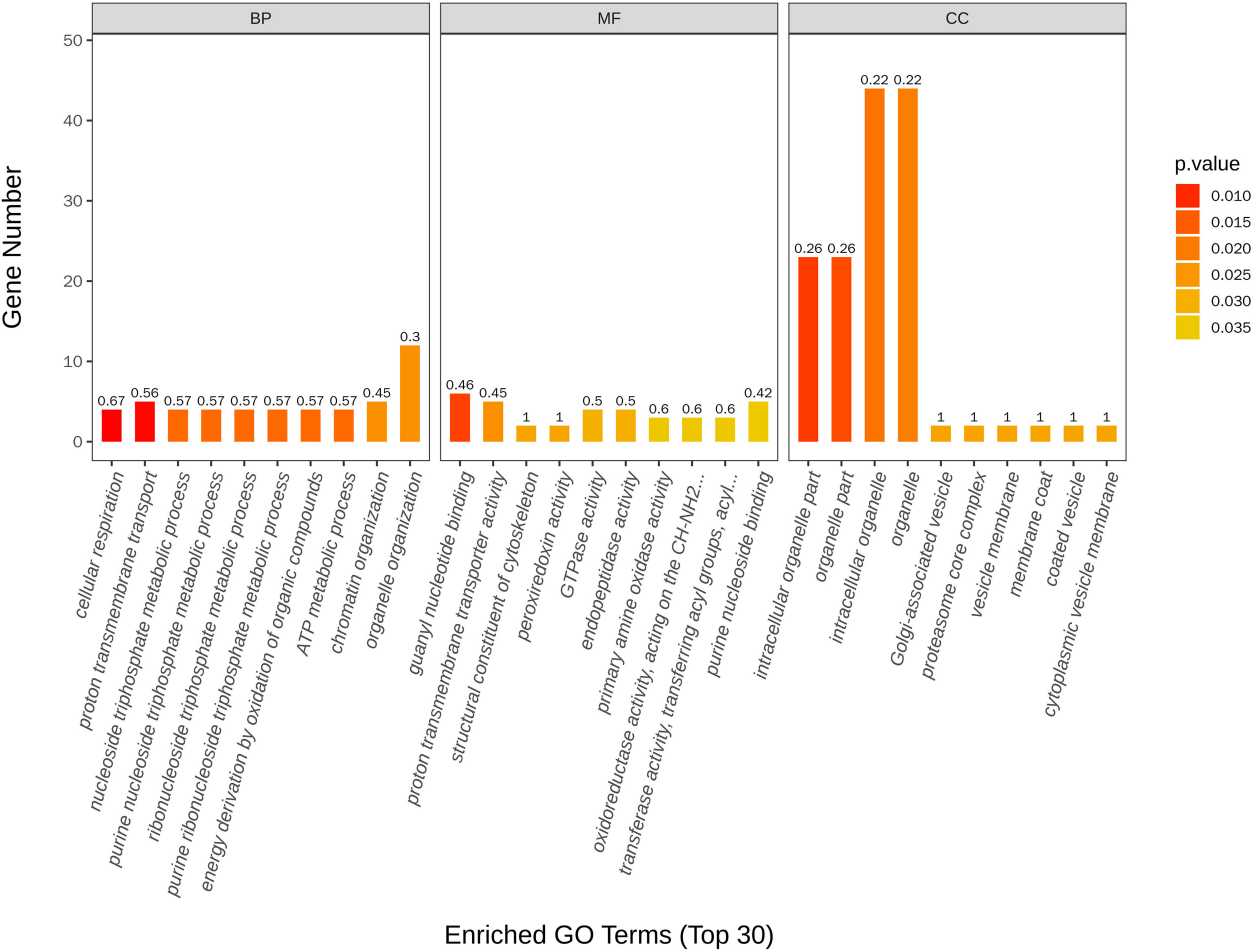
Top 30 GO terms of differentially expressed genes between control and nanoemulsion treatment.

**Figure 8:**
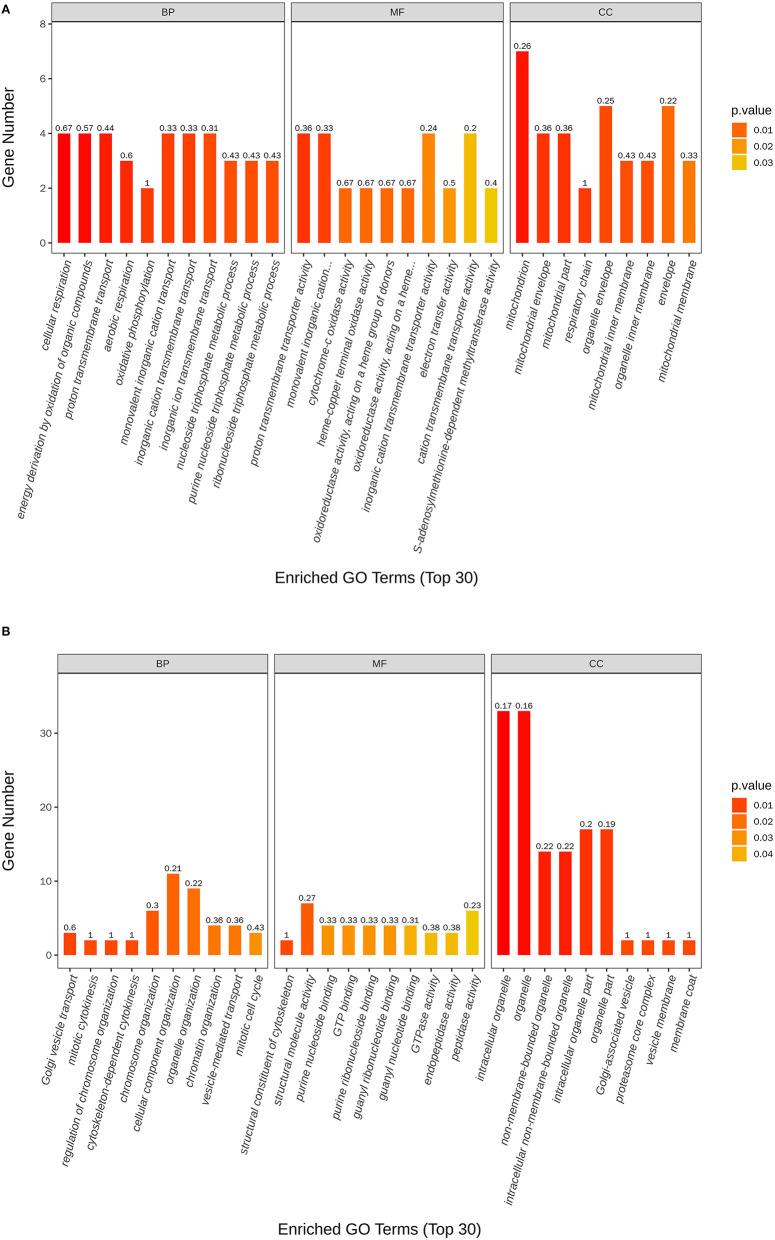
Scatter plots of the top 20 GO enrichment of upregulated **(A)** and downregulated **(B)** DEGs between control and nanoemulsion treatment.

The most enriched GO in 2169 was DEGs (*P*-value < 0.05), which is indicated by Figure 7 and Table 4. In the biological process, the top three significant enrichment terms included cellular respiration (four DEGs), proton transmembrane transport (five DEGs), and nucleoside triphosphate metabolic process (four DEGs). The top three significant enrichment terms in cellular components were intracellular organelle part (23 DEGs), organelle part (23 DEGs), and intracellular organelle (44 DEGs). Furthermore, the top three significant enrichment terms in molecular function were mostly guanyl nucleotide-binding (six DEGs), proton transmembrane transporter activity (five DEGs), and structural constituents of the cytoskeleton (two DEGs). Figures 8A,B shows the top 20 upregulated and downregulated DEGs in GO enrichment (*P*-value < 0.05). The results indicated that the upregulated GO terms were cellular respiration, energy derivation by the oxidation of organic compounds, proton transmembrane transport of biological process; proton transmembrane transporter activity, monovalent inorganic cation transmembrane transporter activity and cytochrome-c oxidase activity of molecular function; mitochondrion, mitochondrial envelope, mitochondrial part of the cellular component of a cellular component. The downregulated GO terms were chromosome organization, cellular component organization, organelle organization of biological process; structural constituent of the cytoskeleton, structural molecular activity, purine nucleoside binding of molecular function, intracellular organelle, organelle, and intracellular organelle part of a cellular component.

**Table 4:** Mostly enriched GO terms of DEGs in *P. digitatum*.

**GO terms**	**Type**	**Input number**	**Background number**	**GO ID**
Cellular respiration	Biological process	4	6	GO:0045333
Proton transmembrane transport	Biological process	5	9	GO:1902600
Nucleoside triphosphate metabolic process	Biological process	4	7	GO:0009141
Purine nucleoside triphosphate metabolic process	Biological process	4	7	GO:0009144
Ribonucleoside triphosphate metabolic process	Biological process	4	7	GO:0009199
Purine ribonucleoside triphosphate metabolic process	Biological process	4	7	GO:0009205
Energy derivation by oxidation of organic compounds	Biological process	4	7	GO:0015980
ATP metabolic process	Biological process	4	7	GO:0046034
Chromatin organization	Biological process	5	11	GO:0006325
Organelle organization	Biological process	12	40	GO:0006996
Nuclear division	Biological process	2	2	GO:0000280
Mitotic cytokinesis	Biological process	2	2	GO:0000281
Developmental process involved in reproduction	Biological process	2	2	GO:0003006
Oxidative phosphorylation	Biological process	2	2	GO:0006119
Regulation of chromosome organization	Biological process	2	2	GO:0033044
Sporulation	Biological process	2	2	GO:0043934
Cytoskeleton-dependent cytokinesis	Biological process	2	2	GO:0061640
Meiotic cell cycle process	Biological process	2	2	GO:1903046
Aerobic respiration	Biological process	3	5	GO:0009060
Golgi vesicle transport	Biological process	3	5	GO:0048193
Amine metabolic process	Biological process	5	12	GO:0009308
Monovalent inorganic cation transport	Biological process	5	12	GO:0015672
Inorganic cation transmembrane transport	Biological process	5	12	GO:0098662
Cell cycle	Biological process	7	20	GO:0007049
Chromosome organization	Biological process	7	20	GO:0051276
Cellular component organization	Biological process	14	52	GO:0016043
Positive regulation of metabolic process	Biological process	4	9	GO:0009893
Positive regulation of cellular metabolic process	Biological process	4	9	GO:0031325
Inorganic ion transmembrane transport	Biological process	5	13	GO:0098660
Intracellular organelle part	Celluar component	23	87	GO:0044446
Organelle part	Celluar component	23	88	GO:0044422
Intracellular organelle	Celluar component	44	199	GO:0043229
Organelle	Celluar component	44	200	GO:0043226
Golgi-associated vesicle	Celluar component	2	2	GO:0005798
Proteasome core complex	Celluar component	2	2	GO:0005839
Vesicle membrane	Celluar component	2	2	GO:0012506
Membrane coat	Celluar component	2	2	GO:0030117
Coated vesicle	Celluar component	2	2	GO:0030135
Cytoplasmic vesicle membrane	Celluar component	2	2	GO:0030659
Golgi-associated vesicle membrane	Celluar component	2	2	GO:0030660
Coated vesicle membrane	Celluar component	2	2	GO:0030662
Cytoplasmic vesicle part	Celluar component	2	2	GO:0044433
Coated membrane	Celluar component	2	2	GO:0048475
Respiratory chain	Celluar component	2	2	GO:0070469
Guanyl nucleotide binding	Molecular function	6	13	GO:0019001
Proton transmembrane transporter activity	Molecular function	5	11	GO:0015078
Structural constituent of cytoskeleton	Molecular function	2	2	GO:0005200
Peroxiredoxin activity	Molecular function	2	2	GO:0051920
GTPase activity	Molecular function	4	8	GO:0003924
Endopeptidase activity	Molecular function	4	8	GO:0004175
Primary amine oxidase activity	Molecular function	3	5	GO:0008131
Oxidoreductase activity, acting on the CH-NH2 group of donors, oxygen as acceptor	Molecular function	3	5	GO:0016641
Transferase activity, transferring acyl groups, acyl groups converted into alkyl on transfer	Molecular function	3	5	GO:0046912
Purine nucleoside binding	Molecular function	5	12	GO:0001883
GTP binding	Molecular function	5	12	GO:0005525
Monovalent inorganic cation transmembrane transporter activity	Molecular function	5	12	GO:0015077
Purine ribonucleoside binding	Molecular function	5	12	GO:0032550
Guanyl ribonucleotide binding	Molecular function	5	12	GO:0032561

### Validation of the Expression of DEGs by qRT-PCR

**Figure 9:**
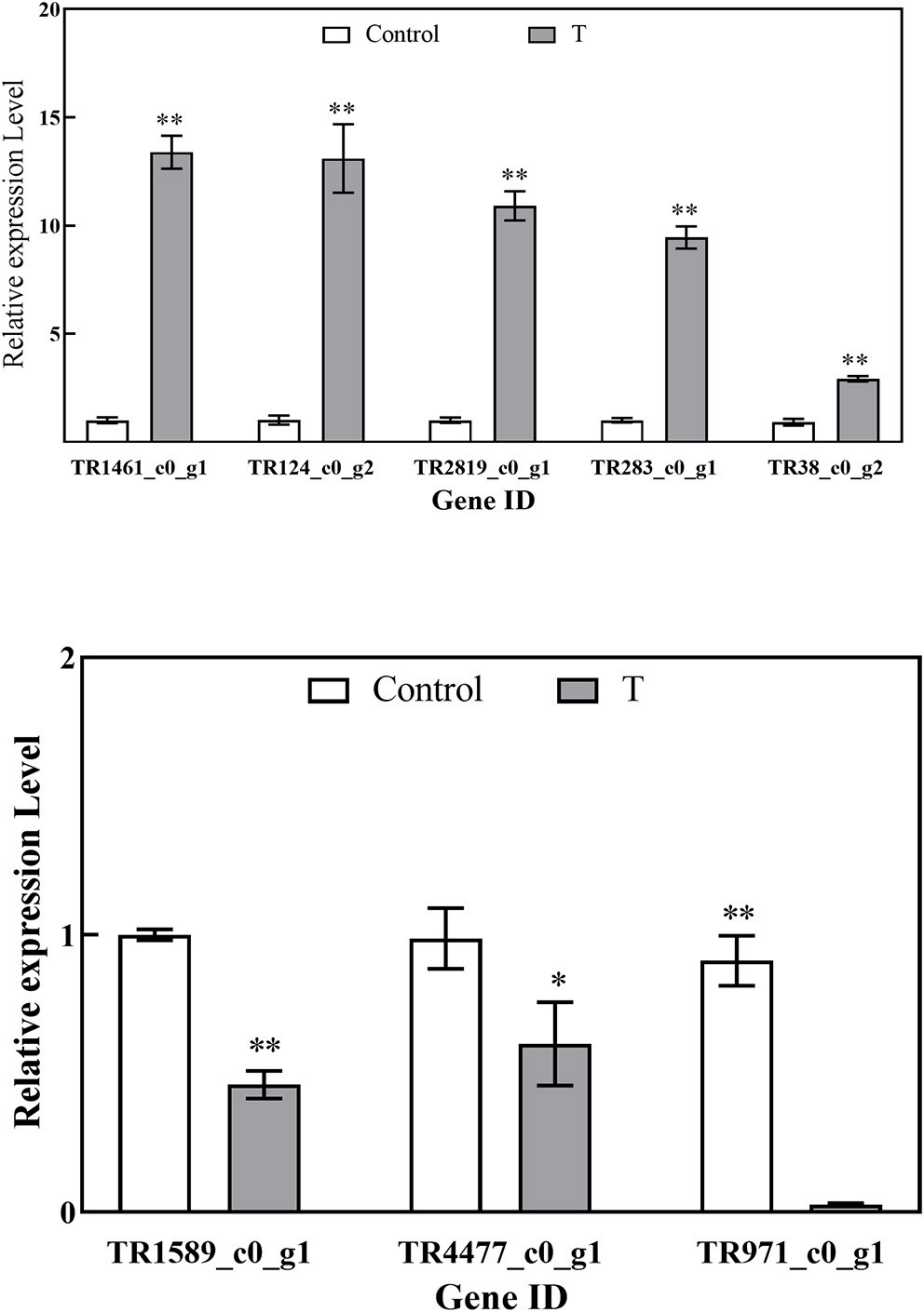
Relative expression levels of DEGs by RT-qPCR. Values are the mean ± standard deviation (SD) of three replicates. The “*”, “**” above the bars on columns indicate a significant difference (*P* < 0.05, *P* < 0.01) compared with control.

As shown in Figure 9, eight DEGs were selected to validate the RNA-Seq results. The results of qRT-PCR experiments indicated that the expression of genes is in line with the RNA-Seq data and then confirmed the reliability of the RNA-Seq data.

### Effect of Nanoemulsion on Membrane Lipid Peroxidation

**Figure 10:**
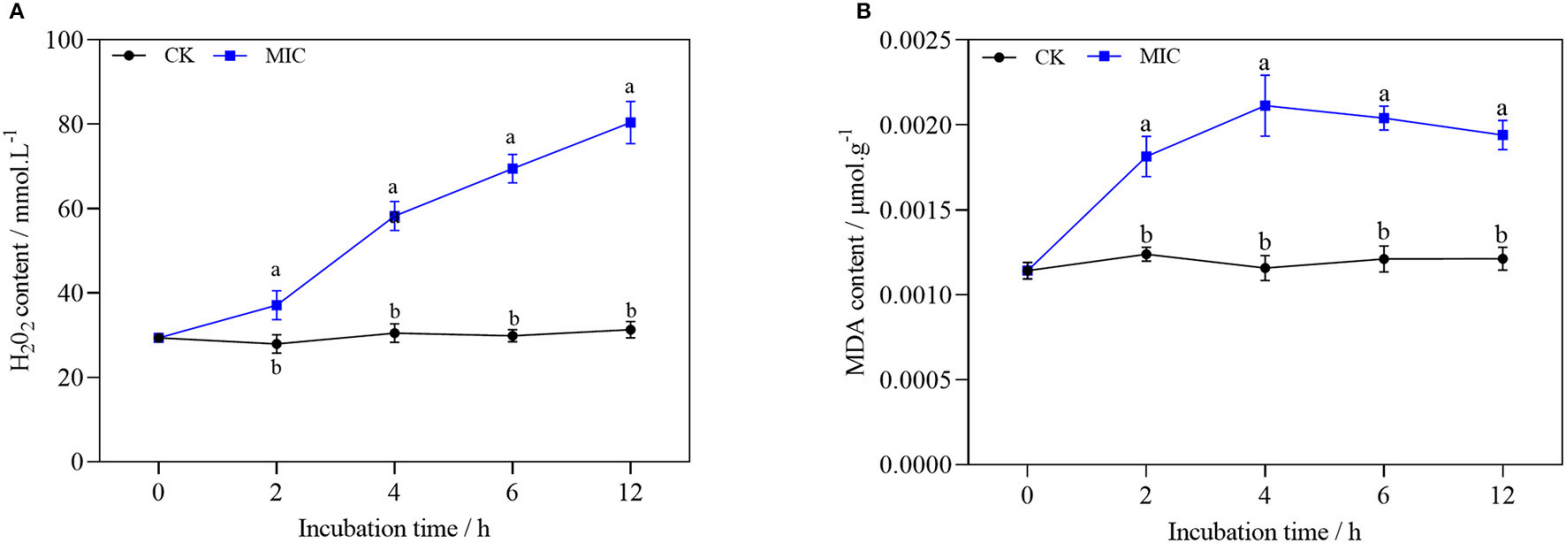
H_2_O_2_ **(A)** and MDA content **(B)** of *P. digitatum* treated without or with MIC nanoemulsion in different incubation times. Values are the mean ± standard deviation (SD) (*n* = 3), and the different letters indicate significant differences (*P* < 0.05).

From Figures 10A,B, after nanoemulsion treatment, H_2_O_2_ accumulation could be significantly induced by nanoemulsion treatment in *P. digitatum*. Compared with the control group, the H_2_O_2_ content of the MIC treatment group increased more than 50% after 4 h. Similarly, the MDA content of mycelia increased significantly, while the content in the control group remained constant. With MIC, the MDA content reached a peak value when treated for 4 h. Afterward, the MDA content decreased gradually in MIC-treated sample, but it was still significantly higher than the control, which implies that the oxidative stress of *P. digitatum* refers to nanoemulsion.

According to previous reports, plant essential oil or constituents can induce eukaryotic cells to accumulate reactive oxygen species, leading to membrane lipid peroxidation damage (33). MDA is one of the essential products of membrane lipid peroxidation. H_2_O_2_ is a kind of reactive oxygen species, and it can be used as a molecular signal to improve cell defense ability and enhance cell tolerance at low concentrations. On the contrary, it can cause oxidative damage of lipid, protein, and nucleic acid molecules at high concentrations (34). After the treatment, the content of MDA and H_2_O_2_ increased sharply, indicating that the nanoemulsion could cause severe damage to membrane lipid peroxidation of the mycelia of *P. digitatum*.

### Effect of Nanoemulsion Treatment on Mycelia Protein Synthesis

**Figure 11:**
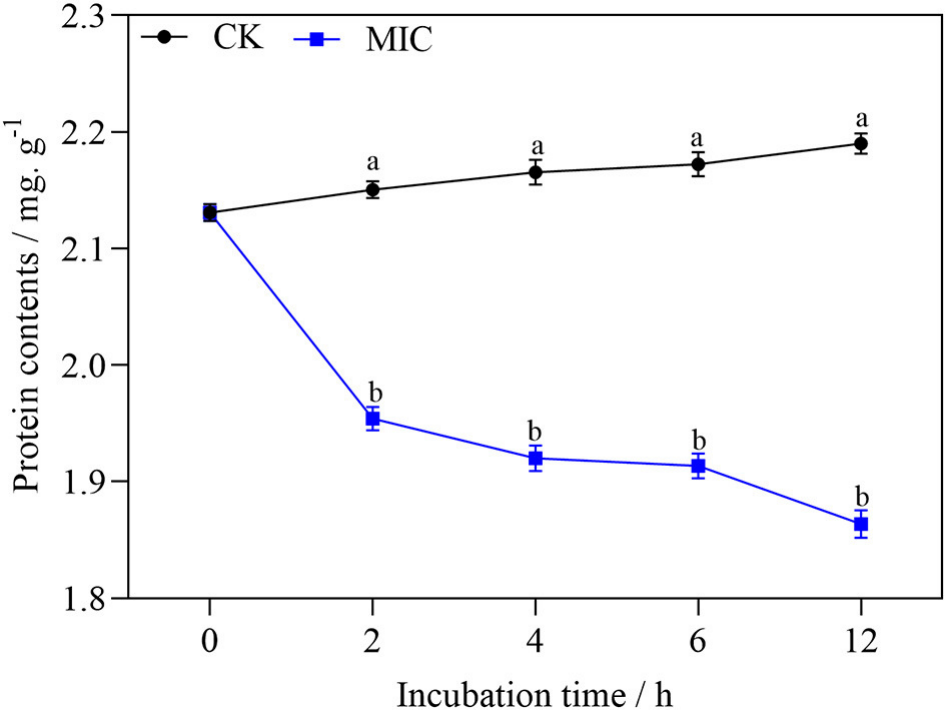
The protein content of *P. digitatum* treated without or with MIC nanoemulsion at different incubation times. Values are the mean ± standard deviation (SD) (*n* = 3), and the different letters indicate significant differences (*P* < 0.05).

As shown in Figure 11, with the extension of treatment time, the soluble protein of mycelium decreased significantly in nanoemulsion samples, while the content of soluble protein in the control group showed a slowly increasing trend. After 2-h treatment, the difference was significant between the treatment and control group. The soluble protein content of nanoemulsion treatment was 1.86 mg/g at 12 h, which is 14.7 % lower than that of the control group, and the difference was significant.

## Discussion

Under the influence of external stimuli, *P. digitatum* cells alleviate the adverse effects on growth by regulating gene expression patterns. Several studies have proved that the antimicrobial mechanism of essential oil includes alternating the distribution of fatty acids in the cell membrane, destroying cell walls, and reducing proton power, and the inactivation of ATPase, amylase, and protease was promoted (35–40). To deeply understand the action mechanism of nanoemulsion from molecular aspects and seek the pathway involved, we used RNA-Seq technology to profile the transcriptome of *P. digitatum* treated with nanoemulsion, which induced the expression of many stress response genes and the pathway involved to alleviate the adverse effect. Recent research by RNA-seq also observed different gene and pathway responses in *P. digitatum* cells treated by antifungal agent [(41, 42)].

### Genes Related to Spore Germination and Cell Growth

Metabolic pathways are critical to cell growth and reproduction, and disruptions in metabolism can lead to cell death (43). KEGG enrichment analysis showed that most DEGs were enriched in the metabolic pathway, there were 144 down-regulated DEGs and 72 up-regulated DEGs respectively, indicating that the nanoemulsion inhibited the growth of *P. digitatum* by reducing its metabolic level. Fatty acids are the energy source for spore germination. The increase in fatty acid and protein contents can promote the germination of spores (44); as indicated by KEGG enrichment analysis, most downregulated DEGs were involved in fatty acid degradation and protein synthesis. Therefore, it can be inferred that nanoemulsion can inhibit spore germination through downregulation of lipid and amino acid metabolism.

Ribosomes are composed of many small subunits and large subunits, each containing a variety of ribosomal proteins and ribosomal RNA molecules. Protein biosynthesis is mainly carried out in ribosomes (45). According to GO enrichment analysis, most DEGs related to ribosome biogenesis in cellular components were downregulated. Four genes (TR4270_c0_g1, TR1973_c0_g1, TR2704_c0_g1, and TR4317_c0_g1) related to the ribosome protein were downregulated 2.73-, 1.80-, 1.71-, and 1.78-fold, respectively, by nanoemulsion compared with control. These genes illustrated that the nanoemulsion treatment destroyed the construction of the ribosome structure of *P. digitatum*.

### Genes Related to Amino Acid Synthesis

Proteins play diverse functions with the cell and are essential for the vesicle trafficking of fungi (46). Protein synthesis is indispensable for spore germination and hypha formation (47). The KEGG enrichment pathway analysis revealed that there were abundant genes that regulate arginine and proline metabolism (13 DEGs); tryptophan metabolism (12 DEGs); phenylalanine metabolism (9 DEGs); tyrosine metabolism (10 DEGs); glycine, serine, and threonine metabolism (12 DEGs); lysine degradation (18 DEGs); and arginine biosynthesis (six DEGs), which were significantly downregulated by nanoemulsion compared with the control. The results are presented in Table 3. According to previous research, fungi usually activate the synthesis of some amino acids to maintain the vitality of cells under adverse stimuli (48, 49). Furthermore, as a signaling molecule, proline can trigger the expression of specific genes, regulate mitochondrial function, adjust osmotic pressure, act as a ROS scavenger, and affect cell proliferation or death (50). In our research, the amino acids and proline metabolism genes were significantly downregulated by nanoemulsion treatment compared with the control, suggesting that nanoemulsion treatment destroyed the osmotic balance ability of *P. digitatum* cells. The soluble protein contents of *P. digitatum* were found to be decreased with increasing treatment time (Figure 8), followed by the downregulation of gene expression of amino acids synthesis in *P. digitatum* revealed by RNA-seq data.

Furthermore, the ubiquitin-26S proteasome system is an important protein degradation system in cells (51) and plays the role of signal transduction, gene transcription, and programmed cell death. We found that 11 genes (TR1589_c0_g1, TR4378_c0_g1, TR4477_c0_g1, TR2443_c0_g1, TR10860_c0_g1, TR2507_c0_g1, TR2739_c0_g1, TR12942_c0_g1, TR1642_c0_g1, TR3076_c0_g1, and TR2492_c0_g1) encoding for 19S regulatory particle and 20S proteasome showed downregulation from 1.07- to 1.95-fold compared with nanoemulsion group with control in our study. Consequently, *P. digitatum* cells produced a huge quantity of damaged and erroneous protein after nanoemulsion treatment. Therefore, we supposed that nanoemulsions inhibit the cell growth by destroying proteins in the cytoplasm and nucleus.

### Genes Related to Cell Integrity

Several studies have proved that the main targets of the antifungal activity of essential oils or their volatile components are cell walls, cell membranes, mitochondria, and intracellular genetic material (52, 53). The cell wall can maintain the cell morphology and control the material transportation and information transmission of the cell. Some proteins in the cell wall also have the function of disease prevention and stress resistance. Filamentous fungi cell walls contain chitin, which plays a pivotal role in the development and pathogenicity of fungi (54). Our results showed that several pathways involved in the cell wall formation, such as amino sugar and nucleotide sugar metabolism, starch and sucrose metabolism, were repressed by nanoemulsion. The gene TR1461_c0_g1 encoded for chitinase expression was upregulated, which might the more chitin was needed for cell wall synthesis, suggesting that the chitin content of *P. digitatum* may increase after the nanoemulsion treatment. The results are similar to the research reported previously (55).

Due to the lipophilicity of essential oils, researchers have proved that the plasma membrane was regarded as the active target of these antifungal agents (56). According to previous research, cells mediated the ratio of saturated fatty acids to unsaturated fatty acids, cis/trans unsaturated fatty acids, and unsaturated fatty acids response to external stress (57). Our data showed that some genes involved in cell membrane compositions were influenced after nanoemulsion treatment, such as biosynthesis of unsaturated fatty acids. Four DEGs in the biosynthesis of the unsaturated fatty acids pathway were all upregulated by 1.19- to 3.24-fold. The above results indicated that *P. digitatum* is responsible for maintaining the fluidity and permeability of the cell membrane by adjusting the composition and content of fatty acids. A similar antifungal mechanism was also reported by the study described by Hu et al. (39). Membrane is the target of *Perilla frutescens* essential oil against *A. flavus*.

Ergosterol is an essential component of the fungal cell membrane. Its primary function is to maintain the fluidity and permeability of the cell membrane. The decrease in ergosterol content usually leads to the disorder of cell permeability and the interruption of cell growth and proliferation (58). Some nanoemulsions encapsulated with antifungal agents showed a promising antifungal activity by inhibiting the ergosterol biosynthesis, leading to cell death (59, 60). In our study, the repressed tendency found in a gene (*ERG2, ERG3, ERG4*) encoding ergosterol biosynthesis was downregulated by 2.62-, 3.57-, and 2.71-fold, respectively. Previous research reported that *ERG3* gene downregulation caused *P. digitatum* cells to lose the capacity to convert lanosterol to ergosterol (61).

### Genes Related to Multidrug Resistance

Under abiotic stress, fungi can reduce intracellular drug levels by activating drug efflux transporters and enhance the ability of exogenous detoxification (62). Fungi drug resistance depends on significant facilitator superfamily transporters (MFS) and ATP-binding cassette (ABC), which can efflux exogenous drugs (63). The gene subfamilies include multidrug resistance (MDR/TAP, ABCB subfamily) and pleiotropic drug resistance (PDR, ABCG subfamily) (64). In our study, two ABCB subfamily genes showed an increase in expression in nanoemulsion-treated samples, which is 3.87- and 1.49-fold, respectively. Five genes belonging to the ABCG subfamily were also upregulated from 2.87- to 11.86-fold. Recent research reported that five transporter genes belong to the PDR network, which can efflux some hydrophobic molecules outside the cell (65), and the PDR proteins play a role in cell sterol uptake (66, 67) and quorum sensing in yeast (68). Therefore, it was speculated that *P. digitatum* could enhance the detoxification capacity and transport nanoemulsion out of the cells to minimize the injuries caused by nanoemulsion to *P. digitatum* cell.

### Genes Related to Stress Response

Fungi could activate corresponding protection mechanisms by adjusting their gene expression under unfavorable external conditions. In our research, gene expression alterations related to stress response were influenced by nanoemulsion. Mitogen-activated protein kinases are present in many eukaryotes, including fungi. It plays an essential role in extracellular signal transduction and cell development and differentiation (69). Research has been reported that there are three classes of MAP kinases, namely, Fus3/Kss1, Hog1, and Slt2 in yeast and filamentous (70), and in the *P. digitatum*, three mitogen-activated protein kinases regulate osmotic pressure, growth and conidiation, cell development, and virulence (71). In our research, one gene, TR3246_c0_g1 encoding MAPK, was downregulated 2.46-fold by nanoemulsion compared with control. These results indicate that nanoemulsion can effectively inhibit *P. digitatum* to pathogenicity-associated MAPK cascades. Research also reported that ΔPdSlt2 mutants generated much fewer conidia than the wild type, indicating that PdSlt2 positively controls conidia formation in *P. digitatum* (72).

Reactive oxygen species is a byproduct of normal oxygen metabolism and decisive in cell signal transmission. Nevertheless, excessive levels of reactive oxygen species can damage cell and gene structure (73). ROS cause damage to biomacromolecules, resulting in devastating damage. DNA may break, mutate, and change its thermal stability after oxidative damage, which seriously affects the standard transcription and translation of genetic information (74). Generally, cells can alleviate ROS damage to cells through the action of enzymes (superoxide dismutase, catalase). Some small molecules, such as glutathione, also play critical cellular antioxidants (75). In our research, the gene (TR16620_c0_g1) relative to peroxiredoxin activity was upregulated by 3.06-fold. The ROS-mediated response has been previously observed for nanoemulsion treatment (76, 77). Similar results showed that genes (*CAT1, SOD1, SOD4, SOD5, AOX2*, and *YHB1*) showed upregulated tendency after peptide MAF-1A treatment, involved in oxidative stress response (78).

Cells can produce a large quantity of energy to neutralize the stress from external stimuli to maintain their vitality. Mitochondria could produce ATP and regulate cell metabolism by oxidative phosphorylation (79). In this research, most genes are related to carbon metabolism, while glyoxylate and dicarboxylate genes related to metabolism were repressed after nanoemulsion treatment. The gene (TR3140_c0_g1) relative to acetyl-CoA acetyltransferase in the tricarboxylic acid cycle and oxidative phosphorylation pathway was downregulated by 2.19-fold, indicating that nanoemulsion treatment might influence the intracellular respiration of *P. digitatum*, which is followed by the most enriched item in the biological process revealed by GO function enrichment analysis of RNA-seq data.

## Conclusions

In conclusion, through RNA-seq technology, we analyzed the effect of nanoemulsion on the *P. digitatum* from the transcriptional level. In-depth RNA-seq analysis revealed that DEGs mainly involved in cell integrity (cell wall and membrane), amino acid synthesis, proteasome, glyoxylate and dicarboxylate metabolism, ribosomes biogenesis, and mitogen-activated protein kinases were notably affected by nanoemulsion treatment. The results indicated that nanoemulsion triggered gene expression variation and induced multiple pathways involvement. A deep understanding of the transcriptomic view mechanism further demonstrated the applicability of nanoemulsion as a natural origination, environment-friendly, and safe approach to combat against the green mold of citrus.

## Data Availability Statement

The datasets presented in this study can be found in online repositories. The names of the repository/repositories and accession number(s) can be found at: SubmissionID: SUB9953553; BioProject ID: PRJNA745208; http://www.ncbi.nlm.nih.gov/bioproject/745208.

## Author Contributions

CW and JC involved in conceptualization, resources, supervision, and project administration. RY, XC, and CC involved in methodology. RY and XC involved in software and formal analysis. RY and CC involved in validation. RY, XC, and QH involved in investigation. RY and CW involved in data curation and visualization. RY involved in writing the original draft preparation. CW and KR involved in writing the review and editing. CW involved in funding acquisition. All authors have read and agreed to the published version of the manuscript.

## Funding

This work was supported by the National Natural Science Foundation of China (31760598) and the Natural Science Foundation of Jiangxi Province of China (20181BCB24005).

## Conflict of Interest

The authors declare that the research was conducted in the absence of any commercial or financial relationships that could be construed as a potential conflict of interest.

## Publisher's Note

All claims expressed in this article are solely those of the authors and do not necessarily represent those of their affiliated organizations, or those of the publisher, the editors and the reviewers. Any product that may be evaluated in this article, or claim that may be made by its manufacturer, is not guaranteed or endorsed by the publisher.
